# The role of live transcripts in synchronous online L2 classrooms: Learning outcomes and learner perceptions

**DOI:** 10.1007/s10639-023-11784-8

**Published:** 2023-04-18

**Authors:** Wang Qiao, Chen Yijun

**Affiliations:** 1 Affiliation: Center for English Language Education, Faculty of Science and Engineering (CELESE), Waseda University, 3-4-1 Okubo, Shinjuku-Ku, Tokyo, Japan; 2 Affiliation: Graduate School of Human and Environmental Studies, Kyoto University, Yoshida-Nihonmatsu-Cho, Sakyo-Ku, Kyoto, Japan

**Keywords:** Synchronous online teaching, Second language classrooms, Live transcripts, Captions, Dual coding theory, Cognitive theory of multimedia learning, Academic reading, Zoom

## Abstract

This study explored the role of live transcripts in online synchronous academic English classrooms by focusing on how automatically generated live transcripts influence the learning outcomes of lower-proficiency and higher-proficiency learners and on their perceptions towards live transcripts. The study adop ted a 2 × 2 factorial design, with the two factors being learner proficiency (high vs. low) and availability of live transcription (presence and absence). The participants were 129 second-year Japanese university students from four synchronous classes taught on Zoom by the same teacher under an academic English reading course. Learning outcomes in this study were evaluated according to the course syllabus through grades and participation in class activities. A questionnaire consisting of nine Likert-scale questions and a comment box was administered to explore participants’ perceived usefulness of, perceived ease of use of, and perceived reliance on live transcripts. Results showed that contrary to previous studies reporting the effectiveness of captioned audiovisual materials in L2 learning, live transcripts as a special type of captions were not effective in promoting the grades of learners of either proficiency. However, it significantly improved the activity participation of lower-proficiency learners, but not that of higher-proficiency learners. Questionnaire results showed that there were no significant differences between learners of two proficiencies in their perceptions towards live transcription, which contradicts previous findings that lower-proficiency learners tend to rely more on captions. Besides enhancement of lecture comprehension, participants reported innovative uses of live transcripts such as screenshots with transcripts for notetaking purposes and transcripts downloaded for later review.

## Introduction

Under the impact of Covid-19, teaching and learning have shifted from traditional classrooms to online learning, and such a situation is likely to persist in the post-pandemic era (Riwayatiningsih & Sulistyani, 2020). In language classrooms, based on sociocultural theories of language learning (e.g., Lantolf & Thorne, 2006), many institutions and teachers are adopting synchronous, or real-time, classes to ensure ample classroom interaction for learning to take place. In such classrooms, online video conferencing platforms are usually required, and the most notable ones include Zoom, Microsoft Teams, etc. As a joint effort to facilitate online learning, such platforms are constantly developing new features for instructional purposes, such as breakout rooms for group discussion and instant polls to solicit answers to questions. One of the recent features relevant to second language (L2) classrooms is live transcription, or real-time captioning, which transcribes speakers’ utterances as they speak. In synchronous L2 classrooms, this feature has the potential to help learners better understand lectures in L2 by providing multimodal input. However, as this feature had not been released until 2021 by many major platforms (e.g., Zoom, 2021 Feb. 24; Chhabra, 2021 Mar. 23), no studies by far have investigated the role of this feature in synchronous L2 classrooms or learners’ perceptions towards the feature. Closely related to this field of investigation, studies on captioned audiovisual materials have revealed overall positive effects of captions on L2 learners’ listening comprehension (e.g., Mirzaei et al., 2017). Nevertheless, it is doubtful whether such results can be generalized to synchronous L2 classrooms with live transcripts, as there are fundamental differences between captioned videos and live transcribed lectures. Therefore, to better understand the role of live transcription in students’ learning outcomes in synchronous L2 classrooms, specific studies are necessary.

## Literature review

### Theoretical underpinnings

The live transcription feature available in current online conferencing tools utilizes automatic speech recognition (ASR) to create speech transcripts in real-time as a speaker speaks. The origin of ASR can be traced back to the 1950s when three researchers in Bell Laboratory built a system called “Audrey” for single-speaker digit recognition (Davis et al., 1952). In the past seven decades, the technology has developed into mature applications in various fields such as human–robot conversation (e.g., Siri) and automated transcription of audio/videos (e.g., Youtube videos). Live transcripts are essentially a special type of caption displayed in an incremental mode, or in the “Karaoke” style: the word or lexical string that is currently heard or uttered is incrementally presented, from one side to another (Lee et al., 2021). Captions are known as uni-lingual (Vanderplank, 1990), intralingual (Williams & Thorne, 2000), or same-language subtitles (Bird & Williams, 2002) or “on-screen text in a given language combined with a soundtrack in the same language” (Markham et al., 2001, p.440). Primarily designed for individuals with hearing disabilities, captions have readily come to be used for language learners who are “hard of listening” since the early 1980s (Vanderplank, 1988, p.272). When captioning was first introduced for use in foreign language classrooms in the 1980s, it was thought to be a way to increase learners’ attention, reduce anxiety, give students instant confirmation of their understanding of what was heard, and increase motivation (Burger, 1989; Froehlich, 1988; Grimmer, 1992; Vanderplank, 1988).

The benefits of captions to L2 learners can be explained by Paivio’s (1986) dual coding theory (DCT) and Mayer’s (2009) cognitive theory of multimedia learning (CTML). In DCT, Paivio (1986) proposed that human memory has two codes, or channels, that deal with visual and verbal stimuli. The information stored in the two channels is linked, which makes information retrieval and processing much easier. DCT is often associated with the cognitive load theory (CLT) (Sweller, 1988), which states that human working memory, or short-term memory, has a limited capacity and that overloading it reduces learning efficiency. Sweller (1988) identifies three types of cognitive load, namely, extraneous load (wasted cognitive effort on unnecessary information), intrinsic load (cognitive effort to understand new information, which varies according to the innate difficulty of information), and germane load (cognitive effort to link new information in the working memory with that in long-term memory). By combining DCT with CLT, Sweller et al. (2011) argued that if learning materials are presented in both visual and aural modes, learners’ cognitive load will be reduced and their working memory capacity will be increased, resulting in improved learning. Building on CLT, Mayer (2009) developed CTML as guidance on how to create effective multimedia presentations for learning. In CTML, three assumptions were proposed: the dual-channel assumption, the limited-capacity assumption, and the active-processing assumption. The dual-channel assumption echoes DCT, and Mayer (2009) specifically pointed out that texts displayed on a screen are also a form of visual information. Following this vein, the use of captioned audiovisual materials in the domain of language learning has received much scholarly attention. Similarly, in the case of live transcripts, more in-depth processing and a higher level of recall of teaching points can be expected, as L2 learners can code the information dually by both listening to and “reading” their teachers’ lectures.

### Captioned audiovisual materials and L2 learning

As it was not until 2021 that mainstream platforms such as Zoom (Zoom, 2021 Feb. 24) and Teams (Chhabra, 2021 Mar. 23) made live transcription a free feature for all their users, no studies by far have investigated the role of this specific type of captions in synchronous L2 classrooms. However, in a closely related field, studies on captioned audiovisual materials for L2 learning are abundant, which may provide empirical evidence for the application of live transcripts in synchronous L2 classrooms.

In recent years, many studies have supported claims that using captioned audiovisual materials can robustly enhance second language learning based on DCT and CTML, and positive results were reported in listening comprehension (e.g., Mirzaei et al., 2017), vocabulary development (e.g., Montero Perez, et al., 2018; Teng, 2019), and reading comprehension (e.g., Muñoz, 2017). However, there have been also studies that suggest captioning cannot contribute to the development of listening comprehension due to such reasons as concentration on reading the text rather than listening to the audio, heavy reliance on the text, overloaded working memory, and learner perception of captions as a source of distraction (e.g., Diao et al., 2007; Hui, 2007; Montero Perez et al., 2013; Taylor, 2005). In these studies, researchers have found that learners’ proficiency levels affect how much they rely on and benefit from captions. In terms of reliance, for example, researchers (e.g., Montero-Perez et al. 2013; Leveridge & Yang, 2014) have suggested that less proficient learners generally rely on on-screen texts more than higher-level learners. While more proficient learners use captions as “a backup to their listening activity” (Pujola, 2002, p.254), low proficiency levels view it as essential for better comprehension. Yeldham (2018) investigated L2 learners’ processing of captioned videos by drawing on the results of nine previous studies, in which L1 and L2 were mostly European languages. The study found that lower-proficiency learners relied more on reading the captions than listening to the speakers whereas higher-proficiency learners in general paid attention to multiple cues simultaneously. In terms of varying benefits for learners of different proficiencies, studies have suggested that captions benefit low-proficiency learners more. Markham (1993), for example, found that captions were only more helpful to advanced learners when the video materials were more abstract or complex. He concluded that for intermediate to advanced learners, captioning should be used only when the video material is difficult for the learners.

Researchers have also compared the effects of different modes of captioning, particularly the effect of full captioning with that of reduced captioning. It is assumed that compared to the plain text in full-captioned videos, highlighted keywords were more salient and thus more likely to be noticed and enter subsequent processing with reduced extraneous cognitive load (Ellis, 2006; Schmidt, 2001). In this vein, Mirzaei et al. (2017) introduced a novel technique called “partial and synchronized captioning” (p.13). The researchers first selected words that were beyond the proficiency level of learners based on hindering factors of comprehension (speech rate, word frequency, and specificity) and the assessed knowledge of the learners. The selected words were shown in captions while the rest were omitted. Then, adopting an incremental display mode, the researchers synchronized texts with audio at the word level. Results revealed that there was no significant difference between the proposed method and full captioning in the comprehension performance of English learners. However, the researchers posited that partial captioning helps decrease learners’ reliance on full captions and hence is better able to prepare them for real-life listening.

### Live transcripts vs. premade captions in videos

Live transcripts resemble premade captions in videos: they provide texts of auditory information and are shown on the screen in consecutive timeframes. However, fundamental differences exist between to two. First, the quality and accuracy of live transcripts are lower than those of premade captions. Captioned videos such as movies and documentaries target a much broader potential audience and are expected to be available for a long time. Thus, those premade captions in videos are carefully checked to ensure quality and accuracy. Meanwhile, for live transcripts, it is commonly acknowledged that ASR tools are rarely 100% accurate, especially in the online environment where technical issues happen frequently. Second, the display modes between the captions in videos and live transcripts are different. Except for the study by Mirzaei et al. (2017) mentioned previously, captions in audiovisual materials in previous studies most appeared in complete lines, while live transcripts are usually shown in word-by-word incremental mode. Third, captions in audiovisual materials are usually limited to one or several lines on the video, while the live transcription feature in major online video conferencing platforms such as Zoom and Teams provide more flexible use of transcripts with a side-by-side transcript panel and download of full transcripts.

In terms of experiment design, studies on captioned videos have primarily examined the immediate effects of captions on learners’ comprehension of those materials (e.g., Hayati & Mohmedi, 2011; Huang & Eskey, 1999). They did not look at how learners’ understanding of those materials would further influence their learning outcomes in a course or performance in an institution-wide unified test. In other words, they focused on the audiovisual materials per se, rather than situating their studies in curriculum goals in L2 classrooms. This also results in a lack of long-term studies. In synchronous L2 classrooms, however, lectures are the target of transcription and therefore the effect of live transcripts should be examined through learners’ learning outcomes in a course. Such an examination naturally requires long-term studies that may span, for instance, one semester or academic year.

The differences mentioned previously mean that the findings in caption-related L2 studies regarding proficiency-specific learner perceptions and learning gains may not fully apply in synchronous L2 classrooms with live transcripts. Therefore, this study sets out to investigate the role of live transcripts in synchronous L2 classrooms by looking at how live transcription influences the learning outcomes of learners of varying proficiencies and to explore learners’ perceptions of this feature in the classroom. The following research questions are therefore proposed:

1. How do live transcripts influence the learning outcomes of learners of varying proficiency levels in a synchronous L2 class?
2. What are learners’ perceptions of live transcripts in the synchronous L2 class?

## Methodology

### Study design

To examine the influence of live transcripts on the learning outcomes of learners with different proficiencies, this study adopted a 2 × 2 factorial design. The two factors were L2 proficiency (low vs. high) and availability of live transcription (presence vs. absence). Thus, four groups of participants were necessary. The researcher decided to situate this study in an English academic reading (AR) course for science and engineering students at a Japanese university. The AR course focused on developing students’ academic reading skills as well as producing written reports to summarize target texts and express their reactions to readings. The reasons for choosing this course were threefold. First, under the impact of Covid-19, online teaching was predominant in Japanese universities when the study was conducted, and the AR course chosen for this study was taught in a synchronous manner through Zoom. Second, the course was taught completely in English, and class time was dominated by teacher instruction, which would maximize the use of live transcripts. Third, the university required students to take the TOEIC test for placement in the course, and therefore, it was easy to find classes that naturally met the grouping requirement of this study.

### Participants

The researchers invited four classes (Class A, B, C, and D) of second-year science and engineering students taught by the same teacher in the AR course to participate in the study. The teacher was a Chinese with native English ability, holds a Ph.D. in second language education and had been teaching the AR course for two years before the study took place. After the exclusion of students with non-full attendance in the course, 129 participants remained in the end (female = 26, male = 103, mean of age = 20.16). Two of them were non-Japanese students (one Chinese and one Korean) while the rest were all Japanese natives. To exclude the possible influence of age and gender, a one-way ANOVA and a Chi-square analysis were performed, and it was confirmed that the distribution of age (F(3,125) = 2.12, *p* = 0.10) and gender (*χ*^2^(3) = 1.06, p = 0.79) did not differ across the four classes. The participants’ placement scores from TOEIC were used to measure their English proficiencies in this study (Class A: n = 32, mean = 499.72, median = 496, SD = 17.48; Class B: n = 34, mean = 610.59, median = 615, SD = 43.98; Class C: n = 33, mean = 493.39, median = 492.00, SD = 28.21; and Class D: n = 30, mean = 615.97, median = 610.00, SD = 31.93). According to the TOEIC-CEFR mapping method (Tannenbaum & Wylie, 2008), participants in Class A and Class C were in higher A2 band while those in Class B and D were in lower B2 band. Welch’s t-tests revealed significant differences in English proficiency between Class A and B (*t*(57.79) = -15.92, *p* = 0.00) and between Class C and D (*t*(43.70) = -13.60, *p* = 0.00), while no significant differences were reported between Class A and C (*t*(53.68) = -1.09, *p* = 0.28) or between Class B and D (*t*(60.22) = 0.56, *p* = 0.58). Therefore, Class A and C were designated as groups of lower proficiency, and Class B and D, groups of higher proficiency. Table 1 shows how the four classes were assigned to the conditions in the study.

**Table 1:** Proficiency and transcription availability of the four classes

	Absence of live transcription	Presence of live transcription
Low proficiency	Class A (L-A)	Class C (L-P)
High proficiency	Class B (H-A)	Class D (H-P)

Note: L = low proficiency, H = high proficiency, A = absence of transcription and P = presence of transcription

### Procedure

Before the study began, the participants signed an online consent form to permit the use of their learning data for research purposes only without any identifying information. They took the AR course weekly for 15 weeks in one semester, with each week’s session lasting for 90 min. The teacher’s lectures were from week 2 to week 14, leaving the first week for orientations and the last for a unified final test. Relevant course materials and activities, including discussion forums, assignments, and tests, were available on the university learning management system (LMS) Moodle.

Each week, the teacher and participants met in Zoom for synchronous lessons. The teacher shared the electronic textbook and the Moodle page on the screen to deliver teaching points and give instructions for in-class activities. For Group C and D, the teacher turned on the live transcripts provided by Zoom, while for Group A and B, this feature was disabled.

### Instruments

#### Live transcription tool

The teacher adopted the default live transcription tool embedded in Zoom, which was developed by the company Otter (https://otter.ai/). The tool enabled adjustment in the position and font size of live transcripts and allowed at most three lines of transcripts to be shown on the screen. According to previous studies on the usefulness of ASR-generated transcripts, a word error rate (WER) of lower than 20% is required for such transcripts to be useful for language learning (Munteanu et al., 2007; Shimogori et al., 2010). Before the experiment, the researchers asked the teacher to rehearse a 10-min lecture on Zoom alone with live transcription on. The researchers corrected the errors in the downloaded transcript and calculated the WER using Amberscript (https://www.amberscript.com/en/wer-tool), a tool specifically designed for calculating WER in automatic transcripts. The result showed that the accuracy of the transcript (1-WER) was 96.70%, higher than the 80% threshold. After the experiment, the researchers did the same with the 26 transcript files (2 classes × 13 sessional transcript files per class) generated during the synchronous classes. The average accuracy in Class C was 95.15% (min = 92.60%, max = 98.20%, median = 94.90%, SD = 1.38%) and that in Class D was 94.80% (min = 91.70%, max = 96.50%, median = 95.00%, SD = 1.51%). A t-test was also conducted between the WER of the two classes and no significant difference was found (*t*(12) = 0.6632, *p* = 0.51). Thus, the default live transcription tool on Zoom was appropriate for this study.

**Fig. 1:**
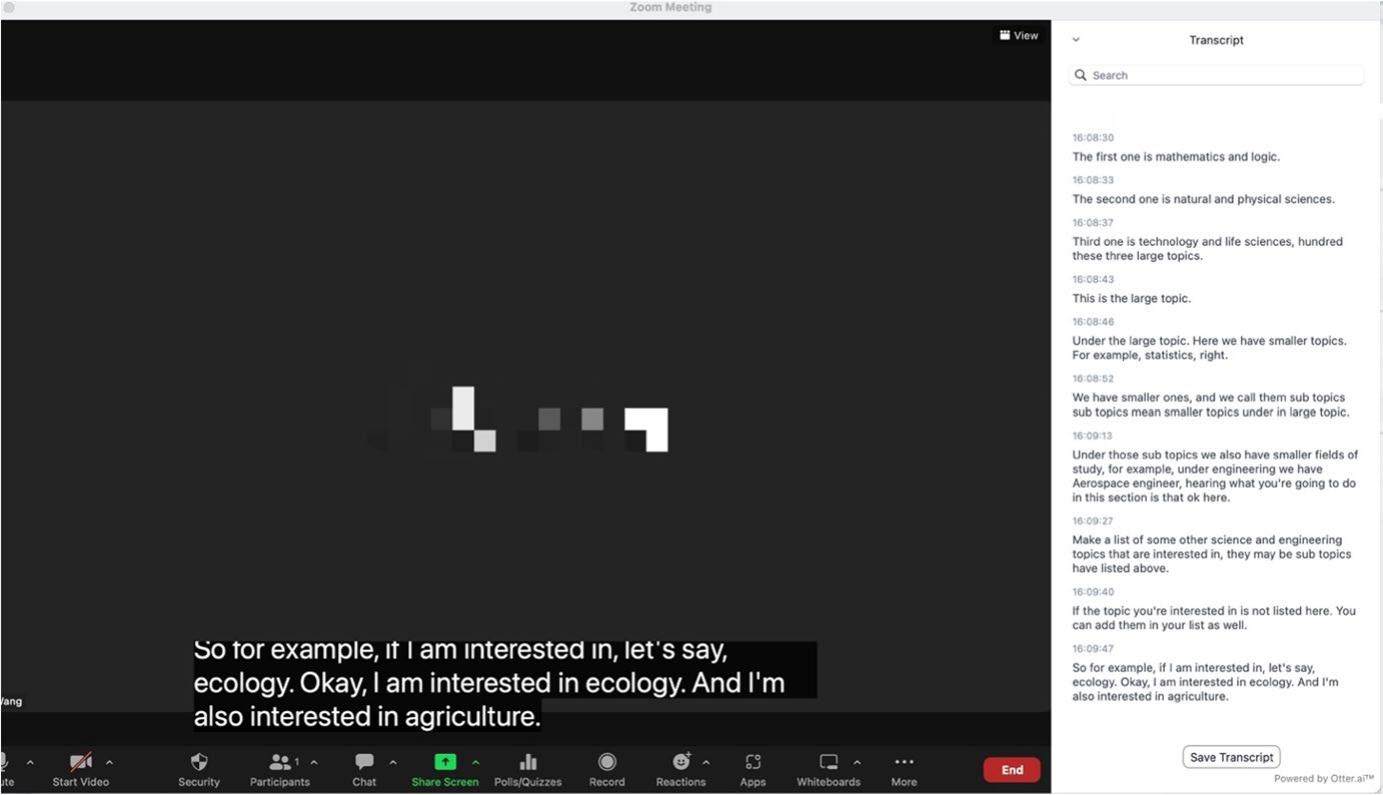
Live transcripts and transcript panel on participants’ screen

During each session in Class C and D, the teacher placed live transcripts at the bottom of the screen, as this was the most accepted location for captions and subtitles in videos, and maximized the font size for participants to see clearly. The participants could choose to hide or show transcripts on their end (by default it was shown) and the teacher asked them to keep the transcripts on for the purpose of this study. Apart from the lines of transcripts appearing at the bottom of the screen, participants on their side could also view the full transcripts with timelines in a panel next to the shared screen if they click the “View full transcript” option (by default, the panel was hidden). They could also download the full transcript for later use. Figure 1 shows the view on the participants’ screen.

#### Evaluation of learning outcomes

Based on the official course syllabus, participants’ learning outcomes were evaluated through graded assignments and tests (grades) and participation in class activities (participants were required to complete at least 2/3 of the class activities on Moodle to get the credit). Accordingly, the researchers decided to investigate the role of live transcripts on grades and participation separately.

Grades in the syllabus consisted of five items: a) five biweekly academic wordlist (AWL) quizzes, b) two reading comprehension tests, c) five summary writing assignments, d) three extensive reading reports, and e) a unified final exam. Among them, the AWL quizzes were designed to test students’ out-of-class vocabulary learning and were not related to teaching in class. Thus, the researcher excluded the vocabulary quizzes from graded items, and the remaining four items added up to 80 points in total.

For class activities, the participants were required to complete in-class exercises by replying to a forum activity on Moodle each week. For example, in the lecture on skimming and scanning skills, the participants were required to write down the main idea of a reading material (skimming) and answer some detail-related questions (scanning) by posting their answers to the Moodle forum that week. The exercises were designed to engage students more actively in the online environment and therefore grading was not necessary. Given the different number of participants in each class, the researchers adopted normalized participation in class activities, or participation rate (the number of activities completed divided by the total number of participants in a session), for participation evaluation. Moodle provides activity completion data for forum activities and the data of the four classes was downloaded to calculate the participation rate in each session.

#### Questionnaire

An online questionnaire (Appendix 1) was administered at the end of Week 14 to understand participants’ perceptions towards the use of live transcripts in the class. The questionnaire adopted the two aspects of Davis’s (1989) Technology Acceptance Model (TAM), namely, perceived usefulness and perceived ease of use, and further included another aspect of perceived reliance, which is a frequent topic in previous studies on captioned videos and language learning (e.g., Yeldham, 2018). Each aspect consisted of three 5-point Likert scale questions in self-reporting format and an optional open-ended question was added at the end of the questionnaire for participants to add comments. Guided by the questionnaire design in Hwang et al. (2013), the researchers focused questions under “perceived usefulness” on how useful live transcripts were in helping participants’ learning; questions under “perceived ease of use” on how easy it was for participants to read the transcripts while attending to other modes of input; and questions under “perceived reliance” on how much attention participants put on live transcripts in class. A reliability test with Cronbach’s alpha was conducted to examine the internal consistency of the questionnaire. The reliability coefficients were 0.86, 0.73, and 0.73 for perceived usefulness, perceived ease of use, and perceived reliance, respectively, indicating acceptable reliability (George & Mallery, 2019).

### Data analysis

This study adopted a mixed-method data analysis approach. Quantitative data included the participants’ grades, participation in class activities, and their responses to the nine Likert scale questions, while qualitative data included their comments in the open-ended question.

For quantitative data, the researchers conducted independent t-tests among the four classes in pairs to examine whether live transcripts promoted the learning outcomes of participants of the same proficiency and whether it helped to bridge the gap in learning outcomes between participants at the two proficiency levels. In terms of the Likert-scale questions, the researchers calculated the mean score of each of the three aspects and conducted a t-test between Class C and Class D to examine if there were significant differences in the responses of participants at the two proficiency levels.

For qualitative data, this study adopted a thematic analysis approach. Two researchers independently coded the participants’ comments in an inductive manner and collaborated to generate themes for the coding results. Any discrepancies in coding were resolved through discussion.

## Results

### Learning outcomes

The following sections show quantitative results on learning outcomes, including grades and participation.

#### Grades

Table 2 and Table 3 show the grading results of the four classes and the t-test results in pairs.

**Table 2:** Grades of participants in the four classes (full score = 80)

Class	N	Min	Max	Median	Mean	SD	SE
A (L-A)	33	48.00	65.00	56.00	55.46	4.25	0.74
B (H-A)	30	51.00	70.00	60.00	59.97	4.38	0.80
C (L-P)	32	45.00	66.00	55.00	55.19	4.96	0.88
D (H-P)	34	44.00	72.00	60.50	59.68	7.26	1.25

**Table 3:** T-test results on grades among the four classes

Comparison pair	t	df	p
A&B	-4.14	60.03	0.00
C&D	-2.95	58.51	0.00
A&C	0.23	60.93	0.82
B&D	0.20	55.16	0.85
B&C	4.03	59.80	0.00

When the availability of live transcripts is controlled, namely, when comparing Class A (L-A) and B (H-A) (mean difference = 4.51, p = 0.00) and comparing Class C (L-P) and D (H-P) (mean difference = 4.49, p = 0.00), results show that higher-proficiency participants significantly outperformed lower-proficiency participants in grades. This supports the selection of the participants based on their placement tests and further proved the validity of subsequent results in this section. When proficiency is controlled, there are no significant differences in grades between Class A (L-A) and Class C (L-P) (mean difference = 0.27, p = 0.82), or between Class B (H-A) and Class D (H-P) (mean difference = 0.29, p = 0.85). This indicates that live transcripts were not able to promote the grades of participants of either proficiency. When neither the presence of live transcripts nor proficiency are controlled, a significant difference is found between Class C (L-P) and Class B (H-A) (mean difference = 4.78, p = 0.00), which means that live transcripts were not able to close the gap between lower-proficiency and higher-proficiency participants in grades.

#### Participation in class activities

Table 4 shows the descriptive statistics on the number of participants completing forum activities and participation rates (in brackets) and Table 5 shows the t-test results.

**Table 4:** Participation in class activities in the four classes

Class	N	Min	Max	Median	Mean	SD	SE
A (L-A)	33	19 (57.6%)	31 (93.9%)	22 (66.7%)	23.231 (70.4%)	3.767 (11.4%)	1.045 (3.2%)
B (H-A)	30	23 (76.7%)	28 (93.3%)	26 (86.7%)	26.077 (86.9%)	1.605 (5.4%)	0.445 (1.5%)
C (L-P)	32	29 (90.6%)	31 (96.9%)	30 (93.8%)	30.385 (95.0%)	0.65 (2.0%)	0.18 (0.6%)
D (H-P)	34	29 (85.3%)	33 (97.1%)	30 (88.2%)	30.385 (89.4%)	1.325 (3.9%)	0.368 (1.1%)

**Table 5:** T-test results on attendance among the four classes

Comparison pair	t	df	p
A&B	**-4.73**	**17.03**	**0.00**
C&D	**4.58**	**18.08**	**0.00**
A&C	**-7.64**	**12.76**	**0.00**
B&D	**-1.33**	**21.94**	**0.20**
B&C	**-5.06**	**15.39**	**0.00**

When the availability of live transcripts is controlled, Class A (L-A) had a significantly lower mean of participation rate than Class B (H-A) (mean difference = 16.52%, p = 0.00), but Class C (L-P) had a significantly higher mean of participation rate than Class D (H-P) (mean difference = 5.58%, p = 0.00). This means that when live transcripts were not available, lower-proficiency participants completed fewer activities than higher-proficiency participants. However, when live transcripts were available, the situation was reversed, and lower-proficiency participants completed more activities. When proficiency is controlled, there is a significant difference between Class A (L-A) and Class C (L-P), but no significant difference between Class B (H-A) and Class D (H-P). This indicates that live transcripts were effective in promoting the activity participation of lower-proficiency participants, but not that of higher-proficiency participants. When neither proficiency nor the presence of live transcripts are controlled, a significant difference is found between Class C (L-P) and Class B (H-A), showing that live transcripts were able to close the participation gap between lower-proficiency and higher-proficiency participants.

### Questionnaire results

#### Likert scale questions

Table 6 and Table 7 present the results on the Likert-scale questions and the t-test results between Class C and D, respectively.

**Table 6:** Results on Likert-scale questions

Aspect	Class	min	max	median	mean	SD	SE
Perceived usefulness	C (Low)	2.00	5.00	4.50	4.15	0.81	0.14
	D (High)	2.50	5.00	4.25	4.20	0.68	0.12
Perceived ease of use	C (Low)	3.00	5.00	4.33	4.13	0.51	0.09
	D (High)	3.00	4.67	4.33	4.13	0.51	0.09
Perceived reliance	C (Low)	3.00	4.50	4.00	4.02	0.39	0.07
	D (High)	2.50	5.00	4.25	4.06	0.63	0.11

**Table 7:** T-test results on Likert-scales questions between the Class C and D

Aspects	t	df	p
Perceived usefulness	-0.28	61.90	0.78
Perceived ease of use	0.05	62.94	0.96
Perceived reliance	-0.30	51.52	0.76

The means of both groups in the three aspects exceeded 4, which indicates that they were generally positive towards live transcripts. Further, there were no significant differences between participants of two proficiency levels in terms of the three aspects. Specifically, participants of higher proficiency slightly exceeded those of lower proficiency in terms of their perceived usefulness of (mean difference = 0.05, p = 0.78) and perceived reliance on (mean difference = 0.04, p = 0.76) live transcripts.

#### Open-ended question

Eight codes were generated from the participants’ responses to the open-ended question, which were further categorized into three important themes, including participants’ reliance on live transcripts, issues with live transcripts, and ways of use. Table 8 is a summary of the coding results.

**Table 8:** Coding results of the open-ended question

Theme	Code	Class C (Low)	Class D (High)	Frequency (Code)	Frequency (Theme)
Reliance on live transcripts	Low reliance	0	5	5	5
	High reliance	1	0	0	
Issues with live transcripts	Technical issues	2	3	5	12
	Difficulties in handling multi-modal input	1	3	4	
	Possible negative effects	0	3	3	
Use of live transcripts	Review	3	3	6	35
	Help with spelling	0	1	1	
	Confirmation of unsure information	6	7	13	
	Complement to limited listening skills	5	13	18	

For reliance on live transcripts, five participants in the higher-proficiency class reported low reliance on live transcripts while one participant from the lower-proficiency class reported high reliance. Exemplary quotes of low reliance included “…but I don’t need the basics”, “I got most information from what the teacher said”, and “It was easy to hear [understand] the teacher’s speech”. The participant with high reliance said, “I used it a lot”. This shows that the teacher’s speech was easy to understand for them, and therefore, some participants tended not to rely much on live transcripts during lectures.

For issues with live transcripts, five participants reported technical issues, including incorrectly transcribed words, the lag between sound and transcription, the small font size on portable devices, and the high scrolling speed of transcripts. Four participants expressed their difficulties in handling multi-modal input: “It is so hard to process [with] my brain”, “However, it may be difficult watch the screen while reading the transcript”, etc. Another three participants unanimously pointed out potential degradation in listening skills caused by too much reliance on live transcripts: “…but too much use will not be good for listening skill”, “It is possible to rely too heavily on live transcripts”, and “To improve our listening skill it is not the best way for students”.

For their use of live transcripts, 18 participants said they used live transcripts to complement their limited listening skills. Examples include: “The live transcript was very helpful because I didn’t understand what the teacher was saying in the English class I was taking before”, “When I could not hear announcement and I could not understand what I do, I looked [at] transcript”, “If I cannot understand the lecture by only the voice, I can realize the content of lecture through live transcripts”, etc. Another 13 participants used live transcripts to confirm information: “when the network environment is poor, [and I cannot hear clearly], I look at the transcripts”, “It was very helpful to be able to confirm from the transcriptions the parts that I did not hear clearly”, etc. More interestingly under “Review”, five participants mentioned their use of the transcript panel to review contents they missed when they were distracted: “Live transcripts were useful when I forgot listening to the lecture and I checked what to do”. Another student innovatively used live transcripts for notetaking, “We can take screen shots at points you [the teacher] think are important”. Lastly, one student mentioned the use of live transcripts for spelling checks: “I was able to check the spelling of what I wanted to write down using the transcripts”.

To sum up, higher proficiency participants reported less reliance on live transcripts. Apart from the well-known use of transcripts to aid listening, participants discovered novel ways of using live transcripts, such as taking screenshots at important points for later review and filling up missing information. For issues of concern, technical problems exist and too much reliance on live transcripts may affect listening skills.

## Discussions 

Results show that the live transcription feature did not promote the grade of participants of either proficiency, but it helped lower-proficiency participants to complete more in-class activities. Results on learner perception show that both groups were positive towards live transcripts, despite some technical issues and the concern over the possible negative effect on listening comprehension. In addition, there were no significant differences between the two proficiency levels in terms of their perceived ease of use of, perceived usefulness of, and perceived reliance on live transcripts, though qualitative results suggest that the higher-proficiency group relied less on live transcripts.

### Ineffectiveness of live transcripts on grades

When the effect of live transcript is examined not through immediate comprehension of audiovisual materials, but through long-term learning outcomes, this study found the grades of participants of neither proficiency groups were improved. There are two possible explanations. First, the benefits of live transcripts on participants’ comprehension of the lectures might have decreased over time. In other words, participants might have benefited from live transcripts in earlier sessions, but as they developed familiarity with the teacher’s speech and accumulated knowledge as the course proceeded, they no longer needed help from live transcripts in later sessions. Similar findings on diminishing or disappearing benefits were also obtained in captioned audiovisual materials in the later stages of long experiments. For example, Rodgers and Webb (2017) conducted a study with 372 Japanese university students who watched ten 42-min episodes of an American television program either with L2 captions or without captions. The authors tested students’ comprehension of the TV episodes through true/false items, multiple-choice items, and sequencing items. The researchers found that in only three of the ten episodes, the students with captioned videos had significantly higher comprehension scores. By the tenth episode, the students with non-captioned videos benefited from their accumulated knowledge and thus the benefits from the captions did not produce a significant difference in comprehension between the two groups.

The second explanation is that comprehension of the lectures did not equate higher grades, which can be further explained by Bloom’s taxonomy of the cognitive domain. According to Bloom (1956), the cognitive domain involves knowledge and the development of intellectual skills, which he categorized into six sequential processes: knowledge, comprehension, application, analysis, synthesis, and evaluation. Among them, participants’ comprehension of lectures in this study falls into “comprehension”, which is related to understanding the meaning or interpretation of instructions or problems and the ability to state a problem in one’s own words (Bloom, 1956). Meanwhile, participants’ grades are more associated with “application”, which is concerned with applying what is learned in the classroom into novel situations (Bloom, 1956), as they needed to use the skills they had acquired through lectures to complete assignments and tests. The sequential nature of the processes means that comprehension, which precedes application, is necessary but not sufficient for application. Thus, the benefits of live transcripts in the comprehension stage were not converted to gains in learning outcomes in the application stage.

### Effectiveness of live transcripts on activity participation

Live transcripts significantly promoted the activity participation of lower-proficiency participants. The researchers believe that live transcripts motivated lower-proficiency participants to be more engaged in the synchronous sessions by enabling them to better understand lectures. Dornyei (1994) described the conditions in learner motivation as Interest, Relevance, Expectancy, and Satisfaction. Among them, Expectancy refers to the perceived likelihood of success, self-confidence, and self-efficacy, on which live transcripts in this study were likely to have had a positive influence. More specifically, live transcripts helped to reduce lower-proficiency participants’ perceived difficulty of the teaching points, or intrinsic cognitive load, by increasing the amount of available assistance and guidance. When lectures were made easier to understand, participants’ expectancy and subsequent motivation to engage in class activities were improved. Previous studies on L2 online learning have also suggested that the difficulty of teaching materials decreases motivation and that students need customized support to be less anxious and uncertain and keep following the course (Chen & Jang, 2010). In exploring challenges facing online learners of the Chinese language, Sun (2014) also found that difficulties in listening to Chinese resulted in the lower motivation of learners.

However, live transcripts did not significantly improve the activity participation of higher-proficiency participants. Based on previous discussions regarding material difficulty/intrinsic cognitive load and motivation, the reason behind the finding may be that higher-proficiency participants were able to understand lectures without the help of transcripts and thus their motivation was not influenced by the availability of live transcripts. This again corroborates previous findings on captioned audiovisual materials that learners of different proficiency levels do not benefit equally from live transcripts. In most cases, learners of lower proficiency levels benefitted more. For example, Guillory (1998) found that captions were beneficial for beginning-level learners. Markham (1993) found that captions were more helpful to advanced learners when the video materials were more abstract or complex. He concluded that for intermediate to advanced learners, captioning should be used only when the video material is difficult for the learners.

### Learner perceptions

Questionnaire results showed that there were no significant differences between lower and higher-proficiency participants in terms of their perceived usefulness of, perceived ease of use of, or perceived reliance on live transcripts. This finding is contrary to those in previous studies that lower-proficiency learners tend to rely more on captions, consider captions more useful but may find it difficult to pay attention to multi-modal input due to the limitations in working memory capacity and cognitive load (e.g., Montero Perez et al., 2013). The reason behind the finding of this study is that live transcripts used in this study were more than a tool to facilitate comprehension in English. They provided participants with more flexible uses. For example, participants used live transcripts to confirm information otherwise unavailable due to network issues, and some took screenshots of lectures for picture-format notetaking. Others used the transcript panel to check instructions they missed due to distraction and downloaded the transcripts for review purposes after class.

For issues with live transcripts, participants reported technical issues and the concern over the potential negative effect on listening skills, which are also important takeaways in previous studies. In Leveridge and Yang (2013), for example, the authors argued that captions must eventually be removed, as the goal of language learning is participation in the target language where captions are not typically available. In terms of the technical issues, when the researchers reviewed the transcripts to calculate WER, they noticed that inaccurate transcription was frequent in spelling students’ Japanese names, sentence segmentation, and recognizing special terms. Previous studies on ASR have also revealed challenges in code switch (Li et al., 2019), automatic boundary detection (Biron et al., 2021), and jargons (Litman et al., 2018).

### Pedagogical implications

Based on the findings of this study, the researchers suggest that teachers who want to apply live transcripts should offer learners strategies in tapping the full potential of live transcripts and help them customize their use in synchronous L2 classrooms. In a previous study, Danan (2004) reviewed the benefits and limitations of audiovisual materials as well as strategies that might optimize the use of captioned materials. The general conclusion was that captions can lead to significant improvement in learners’ listening comprehension as long as they are taught to take advantage of relevant strategies. Thus, before using this tool, teachers should introduce all possible options offered by live transcripts, including the adjustment of font size, the number of lines shown at a time, the transcript panel, and the download of transcripts. In particular, the transcript panel is especially useful in the online environment where learners are more prone to distractions in their surroundings. They can also help learners to decide whether they need transcripts or not depending on their proficiency levels and allow them to switch the transcripts on and off as learners see fit. Particularly, teachers should encourage learners to turn off transcripts in later sessions to avoid overreliance on transcripts. In Hsu’s (2015) study, an adaptive caption filtering mechanism was adopted and tailored to learners’ needs. The results indicated that students would need different amounts of caption support for their listening comprehension according to their preferences. This suggests that the freedom for learners to decide using live transcripts might be more helpful to assist their vocabulary and listening learning. Lastly, if possible, teachers may try paying attention to the transcripts while giving the lecture. When errors occur, they may repeat or rephrase the inaccurately transcribed words.

## Conclusions, limitations, and future studies

This study concludes that live transcripts did not promote the grades of lower-proficiency and higher-proficiency students in the academic reading course, but they increased the motivation of lower-proficiency students to participate in class activities. Besides, there were no significant differences between the two groups regarding their perceived usefulness, reliance, and ease of use of live transcripts. Both groups discovered innovative ways for utilizing the feature apart from enhancing English listening comprehension.

This study is not without its limitations. Design-wise, no questionnaire was administered to participants in classes without live transcripts. Particularly, it would have been better if participants in Class A had been asked why they did not participate in certain activities and how they thought about the difficulty of lectures. Technically, the imperfect accuracy of live transcripts means the information provided to learners was confusing sometimes. This may have undermined the usefulness of live transcripts in the study.

For future directions, researchers who are interested in the role of live transcripts in L2 synchronous classrooms can further investigate how learner control of transcript availability can promote lecture comprehension while reducing cognitive load in processing multi-modal inputs. More broadly, researchers may also explore other latest features provided by online conferencing platforms, such as virtual classrooms that simulate real classrooms by presenting learners’ videos next to one another and virtual avatars where learners’ faces are shown as animated animals, which potentially could encourage learners to keep their cameras on during class.

## Data Availability

The live transcripts generated in this study are available at 10.6084/m9.figshare.21425907.

## Appendix 1

Questionnaire on learner perception towards live transcripts

Please rate on a scale of 1–5 how much you agree with the following statements:

(1: I do not agree at all; 2: I sightly disagree; 3: I neither agree nor disagree; 4: I somewhat agree; 5: I totally agree)

1. Live transcripts helped me understand the lecture.
2. Live transcripts are useful for real-time English classes.
3. I would like to see live transcripts in future real-time English classes.
4. I was able to watch the screen share and read the transcript at the same time.
5. I was able to listen to the teacher and read the transcript at the same time.
6. The live transcript was not distracting at all.
7. I paid more attention to listening to the teacher’s speech than reading the transcript.
8. I paid more attention to watching the screen share than reading the live transcript.
9. I only looked at the live transcript when I did not understand the teacher’s speech.

Please write down any comments you have regarding the use of live transcripts in the class.
